# Representation of genetic association via attributable familial relative risks in order to identify polymorphisms functionally relevant to rheumatoid arthritis

**DOI:** 10.1186/1753-6561-3-s7-s10

**Published:** 2009-12-15

**Authors:** Justo Lorenzo Bermejo, Christine Fischer, Anke Schulz, Nadine Cremer, Rebecca Hein, Lars Beckmann, Jenny Chang-Claude, Kari Hemminki

**Affiliations:** 1 Institute of Medical Biometry and Informatics, University Hospital Heidelberg, INF 305, 69120 Heidelberg, Germany; 2 Division of Molecular Genetic Epidemiology, German Cancer Research Center, INF 520, 69120 Heidelberg, Germany; 3 Institute of Human Genetics, University of Heidelberg, INF 366, 69120 Heidelberg, Germany; 4 Division of Cancer Epidemiology, German Cancer Research Centre, INF 280, 69120 Heidelberg, Germany; 5 Center for Family and Community Medicine, Karolinska Institute, 14183 Huddinge, Sweden

## Abstract

The results from association studies are usually summarized by a measure of evidence of association (frequentist or Bayesian probability values) that does not directly reflect the impact of the detected signals on familial aggregation. This article investigates the possible advantage of a two-dimensional representation of genetic association in order to identify polymorphisms relevant to disease: a measure of evidence of association (the Bayes factor, BF) combined with the estimated contribution to familiality (the attributable sibling relative risk, *λ*_s_). Simulation and data from the North American Rheumatoid Consortium (NARAC) were used to assess the possible benefit under several scenarios. Simulation indicated that the allele frequencies to reach the maximum BF and the maximum attributable *λ*_s _diverged as the size of the genetic effect increased. The representation of BF versus attributable *λ*_s _for selected regions of NARAC data revealed that SNPs involved in replicated associations clearly departed from the bulk of SNPs in these regions. In the 12 investigated regions, and particularly in the low-recombination major histocompatibility region, the ranking of SNPs according to BF differed from the ranking of SNPs according to attributable *λ*_s_. The present results should be generalized using more extensive simulations and additional real data, but they suggest that a characterization of genetic association by both BF and attributable *λ*_s _may result in an improved ranking of variants for further biological analyses.

## Introduction

Susceptibility to rheumatoid arthritis (RA) is determined by both genetic and environmental factors, with an estimated sibling relative risk of 5-10. The *HLA-DRB1 *and *PTPN22 *genes explain approximately 50% of the familial aggregation. A recent genome-wide association (GWA) study carried out by the Wellcome Trust Case Control Consortium (WTCCC) found nine single-nucleotide polymorphisms (SNPs) with associated probability values of less than 10^-5 ^that did not map to loci previously related to RA [1]. In agreement with the common disease-common variant hypothesis, the novel variants were common (minor allele frequencies from 0.07 to 0.41) and they showed genotype relative risks (GRRs) of less than 2.3. The authors of the WTCCC article recognized a disparity between the large population-attributable fraction (PAF) and the modest sibling relative risk (*λ*_s_) explained by the newly identified variants [1]. This disparity is related to the technical characteristics of the platforms used in GWA studies, designed to scrutinize polymorphisms (common variants).

Let us assume that a polymorphism is a marker of a rarer causal variant, and the marker-specific PAF equals the PAF of the causal variant. Under this assumption, we have demonstrated that the causal variant has a higher attributable *λ*_s _than the marker [2]. In other words: for a fixed PAF, causal variants contribute more to familial aggregation (*λ*_s_) than the polymorphisms they are represented by.

There is much debate concerning the representation of statistical evidence in GWA studies. The frequentist *p*-value depends on the power of the study and it does not provide a consistent measure of evidence because identical *p*-values do not imply identical evidence of genetic association [3]. In contrast, the Bayes factor (BF) statistic provides a single measure of the strength of evidence of association.

We hypothesize here that the representation of genetic association by BF together with the attributable *λ*_s _may help to identify polymorphisms relevant to RA, and use simulation and data from the North American Rheumatoid Consortium (NARAC) to evaluate this assumption.

## Methods

### Derivation of attributable risks and Bayes factors

The sibling relative risk (*λ*_s_) attributable to a gene reflects the extent to which a particular genetic locus contributes to familial aggregation. The calculation of attributable *λ*_s _values was first described in an early paper of James [4]. If the frequency of the high-risk allele is denoted by RAF, the relative risk of RA for high-risk allele homozygotes compared to low-risk allele homozygotes by GRR_hom_, the relative risk of heterozygotes compared to low-risk allele homozygotes by GRR_het _and the disease prevalence among low-risk allele homozygotes by *κ*_0_, the attributable *λ*_s _is given by: *λ*_s _= 1+(1/2V_a_+1/4V_d_)/K^2^, where V_a _(additive genetic variance divided by *κ*_0_^2^) equals 2RAF(1-RAF)[(1-RAF)(1-GRR_het_)-RAF(GRR_hom_-GRR_het_)]^2^, V_d _(dominance genetic variance divided by *κ*_0_^2^) equals RAF^2^(1-RAF)^2^[1+GRR_hom_-2GRR_het_]^2 ^and K (population prevalence divided by *κ*_0_) equals RAF^2^GRR_Hom_+2RAF(1-RAF)GRR_het_+ (1-RAF)^2^. Note that if the overall disease prevalence is 50%, *λ*_s _cannot exceed two by definition. However, an important property of the attributable *λ*_s _is that it only depends on RAF, GRR_hom_, and GRR_het_. That is, the attributable *λ*_s _is independent of the disease prevalence among low-risk homozygotes *κ*_0_.

The BF statistic is the ratio of the probability of the observed data under the assumption that there is a true association to its probability under the null hypothesis (absence of association). A small BF provides evidence in favor of a true association. To investigate the relationship between attributable *λ*_s _and the BF, we calculated the expected distribution of genotypes in 1000 cases and 1000 controls when GRR_hom _= 1.5, 2, 3, 4, 5 and 10. Calculations were carried out for the dominant model, which assumes that GRR_het _= GRR_hom_, the recessive model (GRR_het _= 1), and for the additive model (GRR_het _= (1+GRR_hom_)/2). Under Hardy-Weinberg equilibrium, the expected distribution of genotypes in controls (D = 0) is: *Pr*(G = aa|D = 0) = (1-RAF)^2^; *Pr*(G = Aa|D = 0) = 2RAF(1-RAF); *Pr*(G = AA|D = 0) = RAF^2 ^The expected distribution of genotypes in cases (D = 1) satisfies:

GRR_hom _= (*Pr*(D = 1 | G = AA)/*Pr*(D = 0 | G = AA))/(*Pr*(D = 1 | G = aa)/*Pr*(D = 0 | G = aa)) and GRR_het _= (*Pr*(D = 1 | G = Aa)/*Pr*(D = 0 | G = Aa))/(*Pr*(D = 1 | G = aa)/*Pr*(D = 0 | G = aa)). For example, if RAF = 0.2 and GRR_hom _= GRR_het _= 2 (dominance), the expected distribution is (aa = 640, Aa = 320, AA = 40) in controls and (aa = 471, Aa = 471, AA = 59) in cases.

The expected distributions of genotypes were investigated by Bayesian logistic regression. We considered a general three-genotype model of association. Calculation of BFs requires assumptions about effect sizes. We assumed N(0,1) priors on the mean and the two genetic effects. The function *MCMClogit *(R package *MCMCpack *[5]) was used to generate a 10,000 sample dataset from the posterior distribution using a random walk Metropolis algorithm. The *BayesFactor *function in the same package was used to compute log marginal likelihoods for a model 'with' compared to a model 'without' genotypic information using a Laplace approximation.

### Investigated regions

This study investigated regions around the 12 signals detected in the WTCCC study with associated probability values of less than 10^-5 ^(Table 1). The left side of Table 1 represents the 12 selected regions from the WTCCC study. For example, the most associated marker on 1p13 was rs6679677. We retrieved NARAC data from 1000-kb regions centred on the positions of the 12 signals from the WTCCC study. For example, our first region comprised the chromosomal interval (10,901,850::11,901,850) around rs6679677. The WTCCC study reported two SNPs in 6p21 that resulted in two overlapping intervals which were independently investigated.

**Table 1:** Description and results from the 12 investigated regions

WTCCC study^a^					NARAC data^b^			
	
Chr	SNP	Position	MAD of log_10_		Outlying SNPs, gene regions	log_10 _(BF)	RAF	λ_s _Median (5^th^-95^th^)
							
			GRR_hom_	GRR_het_				
1p13	rs6679677	11,401,850	0.282	0.199	rs2476601, *PTPN22*	3.91	0.084	1.048 (1.017-1.098)
1p36	rs6684865	2,578,391	0.282	0.199	-	-	-	-
1p31	rs11162922	80,284,079	0.282	0.199	-	-	-	-
4p15	rs3816587	25,093,513	0.255	0.188	rs12505556, *SLC34A2*	3.04	0.112	1.071 (1.011-1.630)
6p21a	rs6457617	32,771,829	0.306	0.206	rs2395175, *MHC*	16.48	0.148	1.164 (1.108-1.241)
					rs7765379, *MHC*	6.11	0.113	1.175 (1.053-1.668)
6p21b	rs615672	32,682,149	0.306	0.206	rs2395175, *MHC*	16.48	0.148	1.164 (1.108-1.241)
					rs7765379*MHC*	6.11	0.113	1.175 (1.053-1.668)
6q23	rs6920220	138,048,197	0.306	0.206	-	-	-	-
7q32	rs11761231	130,827,294	0.268	0.185	-	-	-	-
10p15	rs2104286	6,139,051	0.290	0.197	-	-	-	-
13q12	rs9550642	19,848,092	0.286	0.200	rs1407961, *ZMYM2*	3.59	0.920	1.033 (1.033-1.225)
21q22	rs2837960	41,433,788	0.288	0.208	rs468646, *MX1*	4.28	0.488	1.038 (1.019-1.057)
					rs466092, *MX2*	3.59	0.488	1.030 (1.016-1.048)
22q13	rs743777	35,876,107	0.283	0.216	rs3218258, *IL2RB*	9.63	0.771	1.061 (1.041-1.084)
					rs710183, *CSF2RB*	4.26	0.072	1.028 (1.011-1.183)
					rs8137446, *KCTD17*	3.48	0.903	1.035 (1.018-1.123)

^a^Results from the WTCCC study.

^b^Results based on NARAC.

### Calculation of attributable risks and Bayes factors for NARAC data

SNPs in the 12 selected intervals were extracted from NARAC data, and the association between RA and the retrieved SNPs was represented by 1) the base 10 logarithm of the BF (log_10_BF) and 2) the attributable *λ*_s_. RA status (affected or unaffected) was modelled by logistic regression, taking into account the covariates gender, number of *HLA*-*DRB1 *shared-epitope alleles (NN = 0, SN = 1 or SS = 2) and genotype (three levels). Individuals with missing information were excluded from the calculations. Only SNPs with the three genotypes represented in both cases and controls were considered.

As already mentioned, calculation of BFs requires assumptions on effect sizes. We calculated frequentist estimates of logistic regression coefficients over the corresponding entire chromosomes using NARAC data. Density plots and tests of goodness of fit of frequentist estimates of log_10_GRR_Hom _and log_10_GRR_Het _for the seven investigated chromosomes indicated that GRR variation was better represented by the median absolute deviation (MAD) than by the standard deviation (data not shown). Therefore, we assumed N(0, MAD^2^) priors on the logarithms of genetic effects. *MCMClogit *and *BayesFactor *[5] were used to calculate BFs for each SNP in the selected regions based on NARAC data. The number of individuals with complete data and the observed allele frequencies in controls were used to draw 10,000 samples from a binomial distribution, which were combined with the posterior estimates of genetic effects to simulate the distribution of attributable *λ*_s _for each SNP.

A bagplot is a bivariate generalization of the non-parametric univariate boxplot [6]. In a bagplot, the region which contains 50% of the observations with greatest bivariate depth is denominated the bag. Observations outside the bag expanded three times are considered outliers, they are too far away from the data's central bulk. These outliers are indicated by closed dots in univariate boxplots. Bagplots have been shown to be equivariant for linear transformations and not limited to elliptical distributions. Due to these properties and the characteristics of our data (unknown shape of the underlying distributions), we used non-parametric bagplots to identify deviating SNPs. Bagplots were determined using the function *compute.bagplot *in the R package *aplpack *[7].

## Results

### Theoretical relationship between Bayes factor and attributable risk

Table 1 shows the RAFs at which the maximum log_10_BF and *λ*_s _were reached assuming several GRR_hom _values under the dominant, recessive and additive modes of inheritance. For example, under GRR_hom _= 2 and dominance, the maximum log_10_BF (25.3) was reached when RAF = 0.22 and the maximum *λ*_s _(1.06) for RAF = 0.17. Alternative sample-sizes/priors would result in different log_10_BFs, but the RAF to reach the maximum log_10_BF would not change. The representation of log_10_BF versus attributable *λ*_s _(data not shown) indicated that the two parameters were correlated, but they did not correspond one-to-one. In the three models, the RAF corresponding to the highest attributable *λ*_s _and the RAF corresponding to the highest log_10_BF decreased with increasing GRR_hom _values. The RAF at maximum log_10_BF was always higher than the RAF at maximum *λ*_s_, i.e., balanced designs resulted in stronger association evidences. The difference between RAF at maximum log_10_BF and RAF at maximum attributable *λ*_s _increased with increasing GRR_hom_, for example (0.24-0.21) = 0.03 if GRR_hom _= 1.5 versus (0.14-0.05) = 0.09 when GRR_hom _= 10 in the dominant model.

**Table 2:** Allele frequencies at maximum *λ*_s _and maximum log_10_(BF)

	Dominant model		Recessive model		Additive model	
			
GRR_hom_	Max log_10_(BF)	Max *λ*_s_	Max log_10_(BF)	Max *λ*_s_	Max log_10_(BF)	Max *λ*_s_
1.5	0.24	0.21	0.70	0.66	0.39	0.40
2	0.22	0.17	0.64	0.61	0.38	0.33
3	0.20	0.13	0.61	0.54	0.33	0.25
4	0.17	0.10	0.59	0.48	0.26	0.20
5	0.16	0.08	0.57	0.45	0.24	0.17
10	0.14	0.05	0.49	0.33	0.17	0.09

### Bayes factors and attributable sibling relative risks in the investigated regions

The analysis of complete chromosomes by frequentist logistic regression resulted in 2(GRR_hom_, GRR_het_) × 8(chromosomes 1, 4, 6, 7, 10, 13, 21, 22) sets of genetic effects. Table 1 shows the MADs of log_10_GRR_hom _and log_10_GRR_het _used as scale parameters in the normal prior distributions. As expected, the *a priori *variance of genetic effects was highest for chromosome 6 (MHC region).

**Figure 1:**
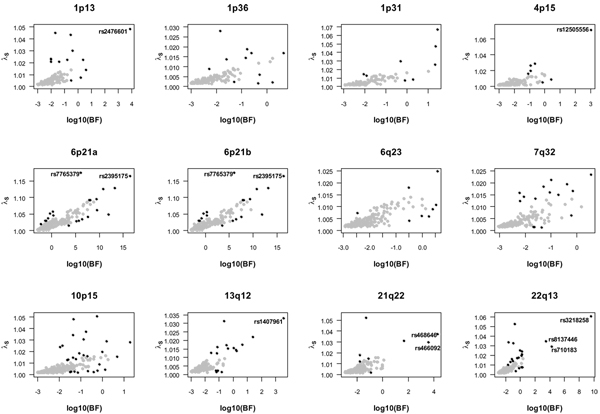
**Scatterplots of log_10 _values of Bayes factors (log10(BF)) and attributable sibling relative risks (*λ*_s_) for SNPs in the investigated regions**. Outlying SNPs (black points) were identified by bagplots. SNP numbers are shown for most extreme outliers with log_10_(BF)s higher than three. Note scale differences.

Figure 1 represents the bivariate distribution of log_10_BF by attributable *λ*_s _based on NARAC data for individual SNPs in the 12 explored regions. Gray points show SNPs within the bulk of data, outlying observations (observations outside the bag expanded three times) are represented by black points. The right part of Table 1 shows SNP numbers and close genes for the most extreme outliers. For example, rs2476601 in the 1p13 region: based on NARAC data, the log_10_BF was 3.91, the high-risk allele frequency in controls is 0.084, and the estimated attributable *λ*_s _with 95% confidence interval was 1.048 (1.017 to 1.098) for this SNP. The other two regions on chromosome 1 did not reveal strong associations with RA. The investigation of the two overlapping intervals in chromosome 6p21 showed practically identical results; the highest attributable *λ*_s _values were found for SNPs rs7765379 (1.175) and rs2395175 (1.164). Many SNPs in this region showed a strong association with RA. The investigation of the 6q23, 7q32, and 10p15 regions resulted in log_10_BF values below three. The log_10_BF and the attributable *λ*_s _for SNP rs1407961 in the 13q12 region clearly departed from the majority of data. Two extreme outlier SNPs were identified in the 21q22 region and three outliers in the 22q13 region.

## Discussion

This study explored the advantage of combining BF, a measure of statistical evidence of association, and the attributable *λ*_s_, a measure of contribution to familial aggregation, in order to characterize the effect of a particular polymorphism on disease risk. Both simulation and NARAC-based results showed that BF usually correlates with the attributable *λ*_s_. Most markers identified in GWA studies confer a slightly increased risk of disease. For this kind of modest genetic effects (GRR ≤ 2), simulation indicated that the maximum log_10_BF and the maximum attributable *λ*_s _were expected to be found for variants with similar frequencies: around 20% (dominant), 40% (additive) and 70% (recessive penetrances). The larger the size of the genetic effect, the lower the RAF to reach the maxima, and the larger the difference between RAFs. Causal variants generally show stronger effects than markers from GWA studies. For example, a recent GWA study identified five novel breast cancer susceptibility loci. The highest GRR was 1.6, which can be compared with GRR = 6 to 30 for carriers of *BRCA1 *mutations. Simulation suggested that, for this kind of strong genetic effects, attributable *λ*_s _and BF are two different dimensions.

The representation of *λ*_s _versus log_10_BF based on NARAC data confirmed that SNPs which have shown replicated associations clearly departed from the majority of polymorphisms in the selected regions. For example, the functionally relevant SNP rs2476601 was unequivocally separated from the rest of SNPs in the 1p13 region. Other genes located in the proximity of the most extreme SNPs were *ZMYM2*, *MX1*, *MX2*, *IL2RB*, and *CSF2RB*. This list includes SNPs which show strong evidence of association (log_10_BF higher than three) and outlying *λ*_s _values. SNPs with high attributable *λ*_s _values but relatively low BFs may also be of interest. Simulation showed that one BF value corresponds to two different *λ*_s _values. In the 12 regions investigated using NARAC data, the ranking of SNPs according to BF differed from the ranking according to attributable *λ*_s_. The difference was particularly clear in the low-recombination MHC region, where rs7765379 showed the maximum *λ*_s _and rs2395175 showed the maximum log_10_BF. This difference in rankings suggests that, in addition to a measure of association evidence, the extensive fine-mapping needed to characterize etiological variants may benefit from the consideration of the attributable *λ*_s_. Future studies based on more extensive simulations and additional real data should investigate this possibility.

The present study made use of Bayesian and robust statistics. The Bayesian approach has been overlooked in the analysis of GWA studies, where the adjustment for multiple testing, the relationship between power and statistical significance, and the selection of disease models are important issues [1,3]. We only considered SNPs with the three genotypes represented in both cases and controls. This strategy would initially hamper the identification of rare causal variants but, on the other hand, logistic regression has not been robust to particular types of outliers, and a three-genotype model with well represented categories would result in more robust estimates. Robust methods aim to describe the structure best fitting to the bulk of the data, and to identify deviating observations. Robust statistics are used a lot less than they ought to be in genetic epidemiology. The use of MADs and bagplots in the present article illustrates their advantage over classical methods in practical situations.

## Conclusion

The association results from GWA studies are usually summarized by a measure of evidence of association (frequentist or Bayesian probability values), which do not reflect the contribution of the identified signals to familial aggregation. We propose here a two-dimensional characterization of genetic association consisting of the attributable *λ*_s _and the BF.

## List of abbreviations used

BF: Bayes factor; GRR: Genotype relative risk; GWA: Genome-wide association; *λ*_s_: sibling relative risk; MAD: Median absolute deviation; MHC: Major histocompatibility complex; PAF: Population-attributable fraction; RA: Rheumatoid arthritis; SNP: Single-nucleotide polymorphism; WTCCC: Wellcome trust case control consortium

## Competing interests

The authors declare that they have no competing interests.

## Authors' contributions

JLB designed the study, performed the statistical analyses and drafted the manuscript. CF and KH contributed to the study design and revised critically the manuscript. AS, NC, RH, LB, and JCC made substantial contributions to interpretation of results. All authors read and approved the final manuscript.
